# Cytotoxicity Comparison of Harvard Zinc Phosphate Cement Versus Panavia F2 and Rely X Plus Resin Cements on Rat L929-fibroblasts

**Published:** 2011-09-23

**Authors:** Sahabi Mahasti, Mandana Sattari, Elham Romoozi, Alireza Akbar-zadeh Baghban

**Affiliations:** 1: Department of Prosthodontics, Dental School of Shahid Beheshti University of Medical Sciences,Dental Research Centre, Tehran, Iran; 2: Department of Immunology, Shahid Beheshti University of Medical Sciences, Tehran, Iran; 3: Department of Radiology, Dental School of Tehran University of Medical Sciences, Tehran, Iran; 4: Department of Biostatistics, Paramedical School of Shahid Beheshti University of Medical Sciences, Tehran, Iran

**Keywords:** Cytotoxicity, Biocompatibility, Resin Cement, Zinc Phosphate Cement, Rat Fibroblast

## Abstract

**Objective:**

Resin cements, regardless of their biocompatibility, have been widely used in restorative dentistry during the recent years. These cements contain hydroxy ethyl methacrylate (HEMA) molecules which are claimed to penetrate into dentinal tubules and may affect dental pulp. Since tooth preparation for metal ceramic restorations involves a large surface of the tooth, cytotoxicity of these cements would be more important in fixed prosthodontic treatments. The purpose of this study was to compare the cytotoxicity of two resin cements (Panavia F2 and Rely X Plus) versus zinc phosphate cement (Harvard) using rat L929-fibroblasts *in vitro*.

**Materials and Methods:**

In this experimental study, ninety hollow glass cylinders (internal diameter 5-mm, height 2-mm) were made and divided into three groups. Each group was filled with one of three experimental cements; Harvard Zinc Phosphate cement, Panavia F2 resin cement and Rely X Plus resin cement. L929- Fibroblast were passaged and subsequently cultured in 6-well plates of 5×10^5^ cells each. The culture medium was RPMI_ 1640. All samples were incubated in CO_2_. Using enzyme-linked immune-sorbent assay (ELISA) and (3-(4,5-dimethylthiazol-2-yl)-2, 5-diphenyltetrazolium bromide) (MTT) assay, the cytotoxicity of the cements was investigated at 1 hour, 24 hours and one week post exposure. Statistical analyses were performed via two-way ANOVA and honestly significant difference (HSD) Tukey tests.

**Results:**

This study revealed significant differences between the three cements at the different time intervals. Harvard cement displayed the greatest cytotoxicity at all three intervals. After 1 hour Panavia F2 showed the next greatest cytotoxicity, but after 24-hours and oneweek intervals Rely X Plus showed the next greatest cytotoxicity. The results further showed that cytotoxicity decreased significantly in the Panavia F2 group with time (p<0.005), cytotoxicity increased significantly in the Rely X Plus group with time (p<0.001), and the Harvard cement group failed to showed no noticeable change in cytotoxicity with time.

**Conclusion:**

Although this study has limitations, it provides evidence that Harvard zinc phosphate cement is the most cytotoxic product and Panavia F2 appears to be the least cytotoxic cement over time.

## Introduction

 Dental cements have a wide range of applications such as serving as luting agents in fixed prosthodontic treatments to enhance tooth-restoration adhesion(1-3).

Zinc Phosphate cements have been the most common luting agents since the early 19^th^ century. Owing to polymerization shrinkage, solubility, low pH and inability of theses cements to establish a chemo-mechanical bond with the tooth, resin cements were introduced into dentistry. These are made up of a major composite resin compartment through which a chemical bond with the tooth is achieved(2-5).

Since resin cements are said to enhance retention of the restorations, they have been increasingly utilized by dentists regardless of their biocompatibility. Also new resin cements such as Rely X Plus and Panavia F2 have been introduced over time(6-9). Cytotoxicity of these materials remains a concern due to the presence of hydroxy ethyl methacrylate (HEMA) and its ability to penetrate into the dentinal tubules(1, 4,10)Given that metal ceramic restorations necessitate extensive tooth reduction, biocompatibility of these products is of concern to avoid pulp necrosis and potential complications associated with failure of these cements. Moreover, in the event that these cements do result in pulp necrosis, restoration removal and root canal therapy would be a challenge for the clinician(4, 11,12,13).Thus, should the cytotoxicity of resin cements be proved, their use should be limited to non-vital teeth. 

Al Fawaz et al.^10^demonstrated that 2 hydroxy ethyl methacrylate and 2-2 bishydroxy methacrylate propoxy phenyl propane can penetrate into the pulp and induce cytotoxic effects on pulp cells. In another study by CetingüÇ et al.^14^HEMA was shown to be present in the pulp cavity of all teeth treated with dentin bonding agents. More recently Schmid-Schwap et al.^1^reported that dual cure resin cements such as Panavia F2 are significantly less cytotoxic compared to other groups of resin cements.

Considering the potential harm associated with resin cements and their cytotoxicity toward pulp cells, further studies need to be conducted to evaluate the biocompatibility of these products. This paper describes an experimental study designed to compare the cytotoxicity of two groups of resin cements (Panavia F2 and Rely X Plus) versus zinc phosphate cement (Harvard) on rat L929-fibroblasts.

## Materials and Methods

### Materials

Cements tested in this study are listed in table 1.

**Table 1:** Test cements and their properties


Cement	Manufacturer	Setting mechanism	Type of cement
Panavia F_2_	Kurary, Japan	Dual cure	Adhesive Resin	
Rely X Plus	3M, USA	Self cure	Resin Ionomer	
Harvard	Haffman,Germany	Chemical(acid-base)	Zinc phosphate	


### Sample preparation

 Hollow glass cylinders with an internal diameter of 5 mm and 2 mm in height were prepared and sterilized with ethylene oxide gas.

### Sample size

 The number of samples for each cement per evaluation time (1 hour, 24 hours and one week) was determined as 10, rendering a total of 90 disks(1,2,3, 4, 5, 6,7, 8) 

### The evaluation intervals

The samples were analyzed in terms of cytotoxicity at 1 hour, 24 hours and one week post exposure to the experimental cements for immediate, acute and delayed chronic reactions respectively.

### L929- fibroblast cell culture

Cell lines of rat gingival L929- fibroblasts were obtained from the Pasteur Institute of Iran. Cells were initially passaged on culture flasks (Passaging: induction of fibroblast proliferation and changing the culture medium). Once an adequate number of cells had proliferated and adhered to the flask, trypsin thylenediaminetetraacetic acid (EDTA) solution (Gibco, Scotland) was applied to detach the cells. These cells were subsequently cultured in 6-well plates at 5×10^5^ cells per 1 ml RPMI_1640 culture medium (Gibco, Scotland). All samples were incubated in 5% CO_2_with humidity > 95%. 

In order to verify the cell viability prior to assessment of cytotoxicity, cells were initially stained with trypan blue dye and observed under light microscope (×40 magnification). For performing (3-(4,5-dimethylthiazol-2-yl)-2, 5-diphenyltetrazolium bromide) (MTT) assay, more than 90%of cells must be vital.

### Negative control

Consisted of cells which were immersed in plates with empty glass discs (without any cement).

### Positive control

Consisted of cells which were immersed in sodium hypochlorite solution and were all expected to die.

### Method

Cements were prepared according to the manufacturers’ instructions. To maintain maximum sterility, all stages of the experiment were performed under a laminar hood. Zinc phosphate cements (Harvard) and the resin ionomer cement (Rely X Plus) were mixed and poured onto the glass discs and allowed to set according to the manufacturers’ recommendations. The discs were subsequently placed into six-well cell culture plates. In the case of Panavia F2 the cement was cured using an Optilux light cure device (Demetron_ Kerr, Danbury, CT, USA; light irradiance 550Mw/ cm^2^) after being mixed and poured onto the discs.

 Each disc was irradiated for 40 seconds according to the manufacturer’s instruction on one side of the disc only. The fibroblast suspension was poured into the six-well plates and then RPMI_1640 culture medium (Gibco, Scotland) plus Streptomycin-Penicillin antibiotics and FBS 10% solutions (Gibco, Scotland) were added to each plate with the discs floating in the solution. The plates were then incubated in a CO_2_ incubator (CO_2_:5%, T: 37.C, W>90%) and subjected to MTT assay for cytotoxicity.

### MTT assay

In this assay the yellow tetrazolium salt (MTT) is reduced in metabolically active cells to form insoluble purple formazan crystals. For this purpose 100 ml of MTT solution (Sigma, USA) were added to each well at three predefined intervals (after 1 hour, 24 hours and one week) and incubated in CO_2_ incubator (CO_2_:5% , T:37℃, W>90%) for 4 hours. After incubation, cells that had survived would reduce MTT and produce formazan resulting in discoloration (Darkening) of the solution. 200 µl of an acid-alcohol solution (Hydrochloric acid/ Isopropanol) were added to each plate after the incubation period and the results were submitted to an enzyme-linked immune-sorbent assay (ELISA) reader (Anthaus 2020, Australia) for analysis of optical density (OD).

### Statistical analysis

 The normality of the distribution of the data was demonstrated using the Kolmogorov-Smirnovtest. Data were analysed using SPSS Version. 13. To evaluate the effect of the cement (Harvard, Panavia F2 and Rely X Plus) and the time (1 hour, 24 hours and one week) simultaneously, two-way ANOVA was used. Cytotoxicity of the different cements was compared regardless of time using one-way ANOVA. Multiple comparisons were performed using honestly significant difference (HSD) Tukey test (p<0.05). Graphs were drawn using Microsoft Excel 2007 software.

## Results

 Cytotoxicity of the different cements at the three intervals is presented in table 2.

**Fig 1:**
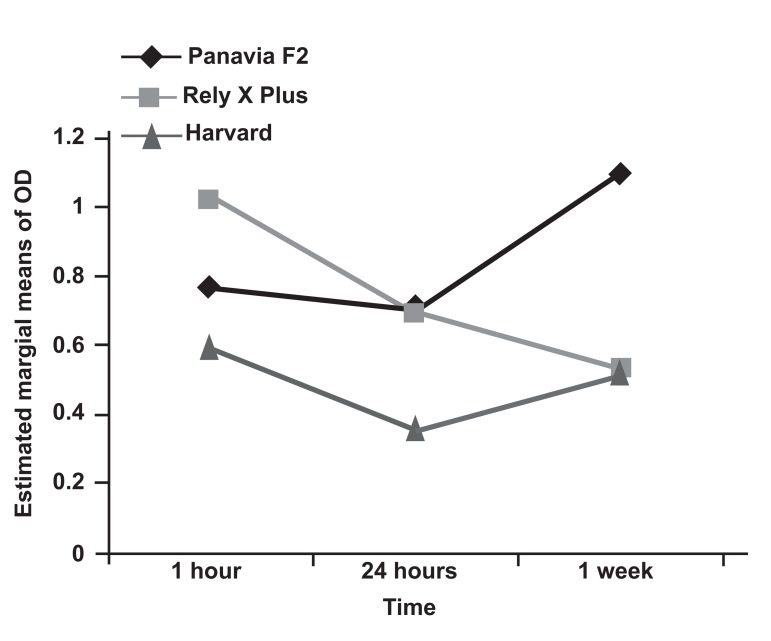
Estimated marginal means of optical density differences for the three cements at the three time intervals.

 Two way ANOVA analysis revealed significant interaction between cement type and time (p<0.001). Figure 1 illustrates that different cements exhibit different degrees of cytotoxicity with respect to time (estimated marginal means of optical density). 

 The results indicate that cytotoxicity differs significantly in Panavia F2 and Rely X Plus cements with respect to time (p<0.001) while this factor did not affect the cytotoxicity of Harvard cement (p≅0.380).

Tukey’s HSD test yielded the following results: 1.In the Panavia F2 group, maximum cytotoxicity was observed after the first hour and the first day. There was no difference between these two intervals (≅0.961); however, the level of cytotoxicity decreased significantly after one week (p<0.001).

**Table 2:** Descriptive statistical indices regarding optical density (OD) of the three different cements at three time intervals


Material	Time	N	Mean	Standard Deviation	95% confidence interval for mean		Min.	Max.
					Lower bound	Upper bound		
Panavia F_2_	1 hour	10	0.769	0.167	0.849	0.888	0.554	1.121	
	24 hours	10	0.705	0.268	0.513	0.896	0.135	1.059	
	1 week	10	1.096	0.875	1.338	2.591	0.578	2.950	
	Total	30	1.015	0.785	0.853	1.439	0.135	2.950	
Rely X Plus	1 hour	10	1.017	0.199	1.031	1.317	0.885	1.450	
	24 hours	10	0.696	0.376	0.426	0.964	0.283	1.452	
	1 week	10	0.534	0.364	0.273	0.794	0.164	1.314	
	Total	30	0.801	0.417	0.645	0.957	0.164	1.452	
Harvard	1 hour	10	0.589	0.243	0.393	0.785	0.412	1.292	
	24 hours	10	0.356	0.080	0.298	0.414	0.240	0.482	
	1 week	10	0.515	0.826	0.070	1.103	0.201	2.858	
	Total	30	0.486	0.496	0.300	0.671	0.201	2.858	


 2.In the Rely X Plus group, maximum cytotoxicity was observed after 24 hours and one week. There was no difference between these two intervals (p≅0.512). Cytotoxicity was significantly less after the first hour (p<0.01) and the first week (p<0.001) respectively.

 To compare the level of cytotoxicity in different cements at different intervals, one way ANOVA was applied. Cytotoxicity differed significantly among the different groups of cements after 1 hour and one week,p<0.001 and after first 24 hours, p<0.05.

### Tukey HSD rendered the following results:

1.Harvard and Panavia F2 cements displayed the highest level of cytotoxicity after the first hour with little difference in toxicity between the two cements (p≅0.176). These levels of toxicity were markedly greater than that observed for Rely X Plus (p<0.001).

2.The highest level of cytotoxicity after 24 hours was observed in the Harvard group. This level of cytotoxicity was significantly higher compared to Panavia F2 and Rely X Plus (p<0.05) (Table 2).

 3.Panavia F2 cement exhibited the lowest level of cytotoxicity after one week. This difference was significant compared to Rely X Plus and Harvard cements (p<0.001) (Table 2).

4.To compare the cytotoxicity of the experimental cements with the positive and negative control, one way ANOVA was utilized.The statistical analysis revealed significant differences between the five groups (i.e. Harvard, Panavia F2, Rely X Plus, positive control and negative control) after 24 hours (p<0.001).

Paired group analysis using the Tukey’s HSD test rendered the following results: the greatest level of cytotoxicity after 24 hours was observed in the positive control group and the Harvard group the difference between which was not statistically significant (p≅0.188).

The lowest level of cytotoxicity was, on the other hand, observed in the negative control group after 24 hours. At this time, Rely X Plus and Panavia F2 exhibited a medium level of cytoxicity with no significant statistical difference between them (p≅0.222,Fig 1).

## Discussion

This study aimed to compare the cytotoxicity of two brands of resin cements (Panavia F2 and Rely X Plus) and one brand of zinc phosphate cement (Harvard) on rat L929-fibroblast. This investigation revealed significant differnces between the cytotoxicity of the different cements at the three intervals (1 hour, 24 hours and one week). The level of biocompatibility after the first hour was in the following order: Harvard<Panavia F2<Rely X Plus. However, after 24 hours and one week, the order changed to: Harvard<Rely X Plus<PanaviaF2.

Schmid-Schwap et al.(1) stated that adhesive resin cements (Panavia F2) exhibit less cytotoxicity compared to self adhesive cements (Rely X Plus) and chemically set cements (Harvard). We also found significantly less cytotoxicity for Panavia F2 than in the two other groups after 1 week. After 24 hours, Panavia F2 and Rely X had less cytotoxic effects than the Harvard cement, but no significant difference between Panavia F2 and Rely X, regarding their cytotoxic effects was observed.

 Ulker and Sengun (4) reported the following order in terms of the cytotoxicity of resin cements:

 Bistite II<Rely X unicem clicker< Panavia F2< Biscem. Different results from the latter research may result from the properties of the Rely X cement. The cement used in the present study was Rely X Plus which is a fluoride releasing resin modified Glass Ionomer with increased cytotoxicity and it's biocompatibility was less than Panavia F2.

Kong et al.(7) demonstrated that Panavia F2, Super Bond C&B and Chemiace II cements induce mild cytotoxic effects on human dental pulp after 72 hours. Panavia F2 in the latter study demonstrsted more cytotoxicity. However, in the present study Panavia F2 proved to be the least cytotoxic cement, which may be due to the different setting mechanisms of the other two cements, Rely X plus and Harvard.

 Schmid-Schwap et al.(1) revealed that dual cure cements (e.g. Panavia F2) were deemed less cytotoxic.

 In another s compared to self cure and chemically cure cements . Study by Bakopulou et al.(11) Rely X displayed significantly greater cytotoxicity on human lymphocytes compared to Panavia F2 where the least cytotoxic cement was shown to be glass ionomere cement. Our findings resemble that of the latter study.

 Franz et al.(8) reported that after preincubation of different cements in the culture media for one week greater cytotoxicity was observed with zinc phosphate cement (Harvard). Likewise, the present study revealed that after one week, Harvard was deemed the most cytotoxic cement. 

Souza et al.(15) studied the effects of resin modified glass ionomer cements on cell cultures and subcutanous tissues in rat. They revealed that all of these cements provoke some evidence of moderate to severe inflammatory response in cells and tissues after 7 days. They also observed that the toxic effect to be proportional to the amount of toxic substances released from these cements and that the amount of cytotoxicity significantly increased with time. Similarly, findings from the present study indicated that the highest level of cytotoxicity in the Rely X Plus group (A resin modified Glass Ionomer cement) was obtained after one week. Moreover, Rely X plus was considerably more cytotxic compared to Panavia F2 after one week and at this time interval is comparable with Harvard cement. Interestingly, in the present study, Harvard cement failed to show significant differences compared to the positive control.

## Conclusion

Although this study has limitations it provided firm evidence that:

1. Harvard cement is probably the most cytotoxic cement and it should be used with caution.
2. Panavia F2 showed the least amount of cytotoxicity after one week compared to Rely X Plus and Harvard.
3. In the Rely X Plus group, cytotoxicity increased with time.
4. Regarding Harvard cement, although the degree of cytotoxicity decreased insignificantly after one week, it is possible that further reductions in cytotoxicity would have occured over a longer time period.
5. Since Rely X Plus exhibits increasing cytotoxicity over time, its use should be limited.

The authors believe that more robust studies are required further to increase our understanding of these cements.
